# Vascular Complications and Diabetes: Current Therapies and Future Challenges

**DOI:** 10.1155/2012/209538

**Published:** 2012-01-09

**Authors:** Abbott L. Willard, Ira M. Herman

**Affiliations:** ^1^Graduate Program in Cellular and Molecular Physiology, Sackler School of Graduate Biomedical Sciences, Tufts University, Boston, MA 02111, USA; ^2^Center for Innovations in Wound Healing Research, Tufts University School of Medicine, Tufts University, Boston, MA 02111, USA

## Abstract

Diabetic retinal complications, including macular edema (DME) and proliferative diabetic retinopathy (PDR), are the leading cause of new cases of blindness among adults aged 20–74. Chronic hyperglycemia, considered the underlying cause of diabetic retinopathy, is thought to act first through violation of the pericyte-endothelial coupling. Disruption of microvascular integrity leads to pathologic consequences including hypoxia-induced imbalance in vascular endothelial growth factor (VEGF) signaling. Several anti-VEGF medications are in clinical trials for use in arresting retinal angiogenesis arising from DME and PDR. Although a review of current clinical trials shows promising results, the lack of large prospective studies, head-to-head therapeutic comparisons, and potential long-term and systemic adverse events give cause for optimistic caution. Alternative therapies including targeting pathogenic specific angiogenesis and mural-cell-based therapeutics may offer innovative solutions for currently intractable clinical problems. This paper describes the mechanisms behind diabetic retinal complications, current research supporting anti-VEGF medications, and future therapeutic directions.

## 1. Introduction

Angiogenesis plays a critical role in the development of diabetic complications, particularly those complications that involve the eye. Diabetic retinopathy is widely considered an inflammatory process [1]. It begins as vascular occlusion and ischemia and may result in macular edema. The eye adapts through vascular proliferation, which can cause blindness through hemorrhage or fibrosis. Complications in diabetic patients are thought to be the pathological result of a single underlying cause, hyperglycemia. Two landmark studies in the 1990s, the Diabetes Control and Complications Trial (DCCT) and the United Kingdom Prospective Diabetes Study (UKPDS), showed that intensive control of hyperglycemia can reduce the occurrence or progression of retinopathy, nephropathy, and neuropathy in both type one and type two diabetics [2, 3]. These findings strongly suggest that hyperglycemia is at the root of diabetic complications. However, strict control of hyperglycemia to the levels outlined in these studies is extremely difficult, and diabetic complications continue to be a significant and sometimes life-ending reality in this population.

As obesity in the United States trends upward, so does the prevalence of diabetes. In 2007, 23.6 million Americans, 7.8%, had diabetes including 23.1% of people older than 60. By 2030, that prevalence is expected to double [4]. For a person with diabetes, the overall risk of death is twice that of nondiabetics at any age. Their average medical expenditure was 2.3x higher than nondiabetics, and total cost was estimated at $174 Billion/year (CDC 2009). 

Diabetes is the leading cause of new cases of blindness among adults aged 20–74 years, and retinopathy causes 12,000 to 24,000 new cases of blindness each year. 25–50% of patients with type I diabetes show some signs of retinopathy within 10–15 years. This prevalence increases to 75–95% after 15 years and approaches 100% after 30 years. 60% of patients with type II diabetes show signs of nonproliferative diabetic retinopathy after 16 years [5, 6].

There is no question that diabetes is a significant individual and public health concern. Uncontrolled hyperglycemia is the foundation from which diabetic complications develop and eventually result in poor health outcomes and quality of life. Although proven to be effective [2], regular examinations and strict hyperglycemic control are difficult to maintain and may result in alternative comorbidities. Therapies that target the specific progression of DR seek to limit the vision loss and other consequences of pathogenic vascular insufficiency.

Currently the gold standard of treatment for PDR and diabetic macular edema (DME) is panretinal and focal/grid photocoagulation. Although this treatment is supported by both the Early Treatment Diabetic Retinopathy Study (ETDRS) and the Diabetic Retinopathy study [7, 8], it seeks only to mitigate the results of the pathogenic process without affecting the underlying cause. Therapeutic interventions that target the pathophysiological mechanism that leads to PDR and DME can be more effective in halting the progression of these complications.

Vascular endothelial growth factor (VEGF) is integral in the angiogenic process and implicated in retinal neovascular development during PDR. Anti-VEGF treatments have been approved by the US Food and Drug Administration (FDA) for treatment of age-related macular degeneration (AMD), and several anti-VEGF medications for DME and PDR are currently in trials. This paper examines the mechanism behind diabetic retinopathy and analyzes the potential effectiveness and limitations of anti-VEGF medications for the treatment of diabetic complications.

## 2. Diabetic Retinopathy

**Figure 1:**
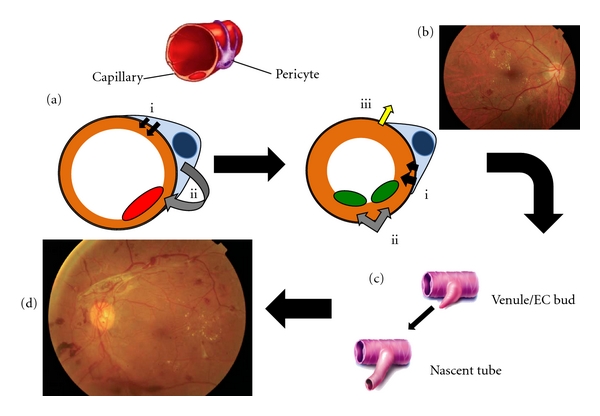
Schematic representation of the progression of diabetic retinopathy. Pericytes interact directly with the normal retinal capillary endothelium (a) within the basement membrane via close contacts and gap junctions ensuring basal tone a(i) and growth arrest a(ii). Persistent hyperglycemia leads to RhoGTPase induction of pericyte contraction b(i) causing reversal of EC growth arrest b(ii) and disrupted matrix contact b(iii) prior to or in the absence of pericyte death/dropout. Basement membrane thickening and leaky, narrow capillaries contribute to thrombosis, ischemia, and the first detectible abnormalities of NPDR. In response to the resultant hypoxia, soluble mediators of angiogenesis, such as VEGF, are released to develop collateral nutrient supply by forming nascent capillary tubes (c). These new blood vessels are highly permeable and fragile and disrupt easily causing hemorrhage and the vision loss characteristic of PDR (d).

Diabetic retinopathy (DR) can be classified into nonproliferative (NPDR) and proliferative (PDR) [9, 10]. The earliest clinical signs of NPDR are microaneurysms and retinal hemorrhaging. Development of cotton-wool spots, venous bleeding, and intraretinal microvascular abnormalities are hallmarks of progressive capillary profusion [5]. PDR is evidenced by the presence of new blood vessels on the surface of the retina and optic disc in conjunction with further retinal ischemia [11]. These new blood vessels become problematic because they are fragile and highly permeable. They break through the optic disc and grow along the surface of the retina and into the scaffold of the posterior hyaloid face. The vessels themselves do not impair vision, but are disrupted easily by vitreous traction and hemorrhage into the vitreous cavity or preretinal space [5]. The neovascularization is also associated with a fibrous component that when contracted can lead to retinal detachment. This stage of PDR poses the greatest threat to vision loss.  Figure 1 shows the schematic representation of the progression of diabetic retinopathy.

### 2.1. Role of the Pericyte

Both NPDR and PDR are thought to be the reaction to increased vascular permeability due to the breakdown of the endothelial-cell-pericyte interaction. The first vascular lesions that occur in the retina are the thickening of the basement membrane, the endothelial injury, the disruption of the tight junctions, and pericyte apoptosis. Pericyte dropout may have profound repercussion on capillary remodeling and will be the main factor responsible for the first abnormalities detected in clinical examination by fundoscopy [35].

Pericytes represent a class of perivascular cells that surround endothelial cells and perform functions on capillaries similar to the ones that smooth muscle performs on arterioles. They are heterogeneous with regard to their origin, distribution, phenotype, and function [36]. Retinal pericytes are associated with the vascular endothelium through extended processes varying in length, arrangement, and form, embedded in the capillary basement membrane [37, 38]. The retina has the highest number of pericytes in the body [39]. This interaction is critical in maintaining the functional integrity of the capillary unit. Pericytes regulate the capillary microenvironment through three principal mechanisms: (1) communication with the underlying endothelium by soluble mediators and cell-cell contact, (2) synthesis, remodeling, and maintenance of the basement membrane, and (3) regulation of microvascular tone through Rho signaling [40]. All of these mechanisms involve an overlapping array of biochemical and biomechanical signaling pathways [41, 42] that are not completely understood.

Pericytes are also intricately involved in vascular development and differentiation. During angiogenesis, nascent microvessels are heralded by an actively motile and proliferative endothelium with an immature basement membrane. This migratory and proliferative phase yields a primitive capillary tube, followed by a microvascular maturation phase marked by endothelial-FGF-2- and PDGF-dependent recruitment of presumptive pericytes, occurring concomitantly with basement membrane remodeling. Triggered by endothelial contact, the presumptive pericyte then assumes a mature contractile status by initiating expression of its smooth muscle contractile protein repertoire [41–43]. Through the subsequent regulation of the capillary microenvironment, pericytes act to suppress endothelial growth [44] and migration [45]. Works done by Hellström et al. and Lindahl et al. suggest that in order for pathological neovascularization to occur, the quiescent endothelium must escape from its growth-arrested phenotype, perhaps actively destabilizing and disengaging from its association with pericytes as it reenters the cell cycle [40, 46, 47]. This is consistent with observations of pericyte dropout seen in pathological states, particularly diabetic retinopathy.

Several pathologies have been indicated that relate hyperglycemia with pericyte dropout and eventual diabetic retinopathy. Initially, it is important to note that pericytes may exhibit dropout though apoptosis and/or migration, both of which lead to the vascular changes that result in DR. Apoptosis is a process of programmed cell death as a result of cellular signaling. It is a very ordered and organized process that minimizes damage to the surrounding tissues. Through specific staining indicative of apoptosis, studies have shown an apoptotic increase in retinal pericytes of isolated retinal capillaries from diabetic patients [48].

However, this does not account for the total extent of pericyte loss seen in experimental DR. Animal models have shown that pericyte apoptosis has been detected after 6 months of hyperglycemia [49] whereas significant pericyte loss is already detectible after three months of experimental diabetes [50]. Pericyte migration has been suggested as an alternative or additional mechanism of pericyte loss. Pfister et al. 2008 show that hyperglycemia-induced pericyte loss predominantly occurs on straight capillaries and is accompanied by increased numbers of pericytes, migrating from the same location into perivascular position [51]. Other studies have shown that pericyte detachment and migration from underlying vessels into the perivascular parenchyma is a feature of pericytes responding to different kinds of stress inducers. For example in brain capillaries, pericytes migrate from capillaries as a result of ischemia, hypoxia, or injury [52, 53]. Most likely pericyte loss includes both apoptosis and migration. As mentioned earlier, pericytes are essential to nascent angiogenesis. Their migration in response to stress may be the physiological initiation of angiogenesis in order to develop collateral nutrient supply. This process is actualized in proliferative diabetic retinopathy as explained later in this paper.

Although the mechanisms for pericyte loss have not been fully determined, several studies have proposed plausible initiation mechanisms that are here described.

**Bax Expression**

Bcl-2-associated X protein (Bax) is a member of the Bcl-2 family. These proteins are well established as inducers and integrators of survival and death signals. The prosurvival family members can inhibit apoptosis induced through cytotoxic insults, and the proapoptotic members generally act through heterodimerizing prosurvival proteins and antagonizing their effects [12]. The relative concentrations of these proteins within the cell are essential for their impact on cell fate. Bax is a known proapoptotic member of the Bcl-2 family.

Studies have shown that Bax overexpression is present in diabetic retinal blood vessels. Specifically, Podestà et al. observed numerous focal increases in Bax staining localized around pericyte nuclei and often associated with TUNEL-positive fragmentation of the same nuclei [13]. They make the association of high glucose, increased Bax, and increased apoptosis, but cannot draw a molecular connection in situ. This data suggests that hyperglycemia increases the levels of Bax in retinal pericytes, tilting the cellular balance of apoptosis regulators in a direction that leads to cell death and eventual retinopathy. More research is necessary to determine the molecular sequence of events.

**TNF-*α***

Tumor necrosis factor alpha (TNF-*α*) is a pleiotropic cytokine involved in the regulation of immune cells during systemic inflammation including initiation of apoptosis. Elevated levels of TNF-*α* have been associated with the early events of DR including the expression of adhesion molecules [14]. Further studies have established the causal link between TNF-*α* and diabetes-enhanced apoptotic loss of microvascular retinal cells. Behl et al. showed inhibition of TNF-*α* caused approximately 76–80% reduction in the number of microvascular cells that expressed apoptotic indicators [15]. TNF-*α* is a known inducer of apoptosis and is shown here not only to be elevated in diabetic retinal microvasculature, but also to be directly involved in cell death.

**TGF-*β***

Transforming growth factor beta (TGF-*β*) is a multifunctional cytokine that regulates signaling pathways. High glucose has been shown to upregulate TGF- *β*1 production in cultured mesangial and glomerular cells [16]. High glucose concentrations can also induce bovine retinal pericytes apoptosis in vitro in association with increased concentrations of TGF-*β* in the culture media [17]. Han et al. show that high glucose levels stimulate pericytes to express TGF-*β*, which in turn upregulates *β*IG-H3 expression, resulting in RGD signaling and induction of retinal pericytes apoptosis [18]. *β*IG-H3 is an extracellular matrix protein that is suggested as being involved in cell growth, [19] cell differentiation, [20] and cell adhesion [21]. It has also been shown to mediate apoptosis through the release of Arg-Gly-Asp (RGD) peptides [22], which in turn activate the caspase signaling cascades in the cell cytoplasm [23]. Here, hyperglycemia upregulates TGF-*β* causing an increase in *β*IG-H3 expression which releases RGD peptides that act through the caspase signaling pathway to induce pericyte apoptosis.

**Ang/Tie**

Tie-2 is a tyrosine kinase receptor primarily found on endothelial cells; however, its RNA has also been reported in pericytes [54]. Angiopoietins, Ang-1 and Ang-2, bind to the Tie-2 tyrosine receptor and have been shown to induce various results. Ang-1 binding to Tie-2 is suggested to mediate the mobilization of hematopoietic stem cells to the peripheral circulation [24] and the formation of mature capillary networks by recruiting periendothelial cells such as pericytes [25]. Ang-2 binding has been suggested to promote smooth-muscle-cell-pericyte dropout, therefore loosening contacts between endothelial cells and periendothelial cells [26]. Cai et al. [27] show that Tie-2 receptors are expressed in retinal pericytes and that Ang-2 protein is upregulated in the diabetic retina as a response to hyperglycemia. They show that Ang-2 plays a role in pericyte dropout and may later be involved with pericyte proliferation in PDR. They also note that Ang-1,2/Tie-2 interactions and downstream effects are dependent on a variety of different factors that also depend on the presence of other cytokines.

Table 1 further highlights the possible causes of pericyte dropout. The mechanisms presented here are comprehensive, but do not include all possible mechanisms. In actuality, the pathway that leads to the eventual loss of pericytes may include some combination of those above as well as others not yet discovered. Also important to note is that the biochemical process that links hyperglycemia and the upregulation of various cytokines has yet to be described.

Alternatively, it has also been suggested that pericyte mechanotransduction may be integral in the development of pathological angiogenesis, negating the need for dropout or death as initiation [40, 55]. Kotecki et al. implicate calpain-dependant cleavage of talin as a critical step in controlling pericyte contractility, perhaps via modulating focal adhesion dynamics. They demonstrate that the strains exerted by pericytes onto substrata can be sufficient to alter normal basement membrane functioning thereby perturbing the endothelial cell mechanical microenvironment [56]. This chemomechanically transduced insult on microvascular contractility could potentially be responsible for endothelial hyperproliferation inherent in diabetic retinal disease.

### 2.2. Angiogenesis in Diabetic Retinopathy

Impaired pericyte function in conjunction with related basement membrane thickening and dysfunction leads to the eventual development of microaneurysms and dot intraretinal hemorrhages. These are two of the earliest abnormalities detectable through ophthalmoscopic examination. Retinal leakage ensues and vasoconstrictive agents lead to a hypoxic environment. Eventually capillaries are constituted only of tubes of thickened basement membrane, and being highly thrombogenic, lead to greater ischemia [35, 57]. The worsening ischemia results in the shift from nonproliferative diabetic retinopathy to proliferative diabetic retinopathy via angiogenesis.

**Platelet-Derived Growth Factor**

Platelet-derived growth factor (PDGF) is a 35 kDa protein originally purified from platelets and now recognized in several cell types including the retina [58]. It acts as an important signaling molecule including several different roles in angiogenesis. Most studied are the hetero- and homodimeric versions, PDGF-AA, PDGF-BB, and PDGF-AB and their structurally related protein tyrosine-kinase receptors [59]. Normal PDGF-receptor interaction assists angiogenesis by enhancing pericyte proliferation and migration most likely through increasing expression of Ang-1 and the resulting signaling cascade [60, 61]. Retinal pericytes have been shown to exhibit the proliferation and chemotaxis in response to PDGF [62], which is also implicated in PDR. Increased levels of PDGF have been identified in the vitreous fluid in patients with PDR [63]. Hypoxia and hyperglycemia, both characteristic of PDR, are shown to increase the production of PDGF in cultured bovine retinal pericytes [64]. PDGF acts as a survival factor in retinal pericytes. Experiencing the hypoxia and ischemia characteristic of diabetic retinopathy, PDGF transcription increases and contributes to angiogenesis. PDGF is necessary for normal retinal vascularization, but its overexpression is a key mediator in the pathogenesis of proliferative diabetic retinopathy.

**Fibroblastic Growth Factor**

Fibroblastic growth factor (FGF) is a family of structurally related heparin-binding proteins known to be potent angiogenesis inducers [65]. It comes in both a basic (bFGF) and acidic (aFGF) form, which act to stimulate endothelial cell migration, proliferation, and microvessel tube formation in cell types that derive from the embryonic mesoderm and ectoderm [66, 67]. FGF in the retina has been reported in ganglion inner and outer nuclear layer, basement membrane of the Muller cells, blood vessels, and retinal pigment cells [68, 69]. Despite its known angiogenic activity and presence in retinal cells, the pathologic activity of FGF in DR has yet to be determined.

**Hepatocyte Growth Factor**

Hepatocyte growth factor (HGF) is 90 kDa cytokine synthesized in the liver that is active in regulating cell growth, cell motility, and morphogenesis of various types of cells [70]. It acts on epithelial and endothelial cells through a paracrine interaction with a high affinity c-Met tyrosine-kinase surface receptor [71]. High concentrations of HGF have been recognized in serious PDR with coexisting fibrovascular proliferation [72]. Although present in retinal cells, the linking mechanism to PDR and conclusive evidence have not been discovered.

**Role of VEGF**

In addition to the factors described above, vascular endothelial growth factor (VEGF) plays a critical role in both physiological and pathological angiogenesis. The human VEGF gene is organized into eight exons and is localized in chromosome 6p21.3. Alternative exon splicing results in the generation of four main isoforms having 121, 165, 189, and 206 amino acids, respectively. VEGF_165_ is the predominant molecular variant as a heparin-binding homodimeric glycoprotein of 45 kDa [73–75].

VEGF exerts its action through two high-affinity tyrosine kinase receptors, VEGFR-1 (Flt-1) and VEGFR-2 (Flk-1). Evidence suggests that VEGFR-2 is the major mediator of the mitogenic, chemotactic, angiogenic, and increased permeability effects of VEGF [76]. This ligand receptor interaction has been shown to stimulate microvascular endothelial cell proliferation [76] and EC migration [77], inhibit EC apoptosis [78], and induce angiogenesis [79].

Downstream effects of the upregulation of VEGF include increases in proinflammatory mediators like intercellular adhesion molecule 1 (ICAM-1). Increased presence of ICAM-1 is associated with leukostasis and vascular permeability recognized in animal diabetic retinopathy [80–82]. These findings substantiate the significance of inflammation in the progression of DR.

There are several mechanisms that have been shown to participate in the regulation of VEGF gene expression. Of particular significance in the pathogenesis of PDR is the upregulation of VEGF in response to hypoxia. VEGF mRNA expression is rapidly and reversibly induced by exposure to low pO_2_ in a variety of pathophysiological circumstances [83, 84]. This is mediated through hypoxia-inducible factor 1 (HIF-1) [85]. HIF-1 is a basic, heterodimeric, helix-loop-helix protein, transcription factor composed of the constitutively expressed HIF-1**β**, and the oxygen-sensitive HIF-1**α**subunits [86]. In appropriately oxygenated tissues, HIF-1*α* is broken down. In hypoxic conditions, the breakdown of HIF-1*α* is inhibited and it begins to build up and dimerize with HIF-1**β*. *This complex then binds to DNA, helps to recruit coactivators, and activates transcription of its target genes, including VEGF [87, 88].

As a survival response to the ischemic environment created by NPDR, transcription of VEGF increases, leading to endothelial cell proliferation, progenitor cell migration, and pathological angiogenesis. Increased intravitreal levels of VEGF have been shown in patients with DME and PDR [89], implicating VEGF as a significant contributor to the pathogenic process and an important focus for intervention.

## 3. Anti-VEGF Treatments

There are four anti-VEGF pharmacologic agents commercially available and in trials for their use as treatment for DME and PDR. Pegaptanib (Macugen, OSI/Eyetech, Melville, NY, USA) is a pegylated aptamer that targets VEGF_165_, inhibiting its endothelial mitogenic activity and vascular permeability effects [90, 91]. Based on the results of the VEGF Inhibition Study in Ocular Neovascularization (VISION) trial, the FDA-approved Macugen for the treatment of neovascular AMD [92]. Although shown to be effective, pegaptanib therapy has been replaced by the development of nonselective anti-VEGF therapies.

Both bevacizumab (Avastin; Genetech, San Francisco, CA, USA) and ranibizumab (Lucentis; Genetech) are humanized monoclonal antibodies, full length and fragment, respectively, that bind all VEGF isoforms. The FDA has approved Lucentis for neovascular AMD, and Avastin is being used off-label for treatment of various ocular diseases.

The final anti-VEGF treatment is the VEGF Trap-Eye (Regeneron Pharmaceuticals, Inc., Tarrytown, New York, NY, USA and Bayer Healthcare Pharmaceuticals, Berlin, Germany). It is a 115 kDA recombinant fusion protein consisting of the VEGF-binding domains of human VEGFR-1 and VEGFR-2 fused to the Fc domain of human immunoglobulin-G1 [93]. It acts to sequester VEGF, binding preferentially over the physiological receptors. Animal studies have shown intravitreal VEGF Trap-Eye has theoretic advantages over other anti-VEGF treatments, including longer half-life in the eye and a higher binding affinity to VEGF-A and related proteins, placental growth factors 1 and 2 [94, 95]. Trap-Eye is currently in phase two trials.

### 3.1. Clinical Trials and Safety Implications

Recently published prospective studies of anti-VEGF medications for both DME and PDR show the clinical effectiveness of these medications and point to possible adverse events. Studies that focused on the effectiveness of pegaptanib in DME come from the work of the Macugen Diabetic Retinopathy Study Group [28]. The study involved 172 patients, randomized into four arms: 3 mg intravitreal pegaptanib, 1 mg, 3 mg, or sham given at weeks 0, 6, and 12. Eyes in the pegaptanib group showed more improvement over the sham eyes, particularly in the 0.3 mg group. The improvement included better visual acuity, more reductions in central retinal thickness, and less need for macular laser photocoagulation [96]. These study patients had no previous history of treatment for DME, and subsequent studies have shown that treatment-naïve eyes respond better to anti-VEGF therapy [32, 97]. Trials investigating the effectiveness of pegaptanib for the treatment of PDR have yet to be published.

A recent trial of ranibizumab has shown its effectiveness for the treatment of DME. Nguyen et al. conducted the ranibizumab for Edema of the Macula in Diabetes (READ-2) Study to investigate treating DME [98]. The group randomized 126 eyes into three groups: the first received 0.5 mg intravitreal ranibizumab at baseline and months 1, 3, and 5; the second underwent focal/grid laser photocoagulation at baseline and again at three months if needed; the third group underwent laser at baseline and ranibizumab injections at baseline and 3 months. Their primary endpoint was an improvement in best-corrected visual acuity (BCVA) at six months then throughout a follow-up period lasting to 12 months. At six months, the first group showed the largest statistically significant improvement in BCVA, +7.24 letters versus −0.43 and +3.80 letters for groups 2 and three, respectively. After two years the first group still showed the largest improvement in BCVA; however, there was little change between the six-month and two-year results. The authors attribute this lack of change to the heterogeneity of patients with DME and note that the post-six-month regime may not have been ideal for all patients [98]. They suggest that perhaps some combination of more frequent injections or greater amounts will provide better control over edema.  Table 2 further describes the considerable current clinical activity regarding the role that anti-VEGF therapies may play in DME.

Bevacizumab is, for both DME and PDR, the best studied anti-VEGF medication due to availability and a cost difference of US$150 versus US$1600 for a dose of ranibizumab [99]. Nicholson and Schachat review the clinical trials and results of bevacizumab for both DME and PDR [96]. They note beneficial short-term effects on treatment of naïve eyes for DME and laser-refractory DME including improvements in both visual acuity and central macular thickness. In trials pertaining to PDR, treatment with bevacizumab has been promising in areas of decreasing leakage in neovascular lesions, showing a substantial effect on new vessels. Mirshahi et al. conducted the largest of the clinical studies in patients with bilateral PDR with high-risk characteristics [100]. In one eye they received 1.25 mg bevacizumab injection with scatter laser treatment according to the ETDRS protocol and the other eye received a sham injection with the scatter laser treatment. After six weeks, almost 90% of the intervention eyes had complete regression of neovascularization versus 25% in the sham group. Although remarkably effective, the results were short lived and there was no difference in the two groups after 16 weeks. Other reviewed trials show similar results. Most notably absent from the available clinical trials are long-term, well-designed studies that involve multiple dosing and comprehensive investigation of potential adverse events. This may be due to a lack of appropriate study techniques.

The VEGF Trap-Eye is the least tested anti-VEGF treatment. The DME and VEGF Trap-Eye: Investigation of Clinical Impact (DA VINCI) study compares differing doses of the intervention with standard macular laser treatment [101]. All Trap-Eye treatment arms of the study showed significantly better mean visual acuity outcomes and greater mean reductions in retinal thickness over the 24-week period. These results are similar to those shown by the three other treatment interventions although no direct comparisons have been performed.

Each therapy seems to show some beneficial effect to treatment endpoints like best-corrected visual acuity and central macular thickness, but an investigation of safety and adverse events taking into consideration the limitations of current trials is essential.

Much of the safety information from these interventions comes from their use and study of age-related macular degeneration. These studies are generally ineffective and underpowered at detecting adverse events, and larger prospective trials with methodological surveillance are still needed. For pegaptanib, the VISION trial attributed no systemic side effects to treatment over the course of the study [92]. For ranibizumab, the ANCHOR [102, 103] and FOCUS [104, 105] trials reported small rates of endophthalmitis and no serious nonocular adverse events. Pooled data from the MARINA, ANCHOR, and PIER studies showed a slightly increased rate of vascular events, which may be clinically irrelevant given the 2-year safety data [96].

As before, studies involving bevacizumab yield the largest amount of information. A large retrospective study of 1,173 patients who received intravitreal bevacizumab yielded systemic adverse events: seven cases of acute hypertension, six cerebral vascular accidents, five myocardial infarcts, two iliac artery aneurisms, and two toe amputations. There were several ocular adverse events: seven cases of endophthalmitis, seven cases of tractional retinal detachment, four cases of uveitis, and a less significant representation of others [106]. These results are similar to those found from other bevacizumab studies and those of other anti-VEGF agents. Some additional systemic effects have been recognized in bevacizumab use in cancer therapy including arterial thromboembolism, gastrointestinal perforation, hemorrhage, hypertensive crisis, and nephritic syndrome [107, 108]. An additional study also showed that bevacizumab when used systemically with chemotherapeutic agents can increase the risk of thromboembolic events twofold over chemotherapy alone [109].

Other adverse events important to note are the development of tractional retinal detachment (TRD) and the possibility of systemic drug entry. Clinical trials of bevacizumab for treatment of PDR show increased risk of TRD following a rapid retinal neovascular regression [110–112]. Further, Avery et al. found decreased neovascular leakage in untreated “fellow” eyes raising the possibility that after injection of 1.25 mg, systemic inhibitory concentrations are achieved [113]. As systemic anti-VEGF therapy may be responsible for the increased risk of thromboembolic events seen in cancer therapy, an unintended trespass across the blood-retinal barrier may have far-reaching consequences.

## 4. Discussion and Conclusions

The diabetic retinopathy cascade begins with chronic hyperglycemia affecting the normal progression of glycolysis. The downstream effect includes pericyte dropout and/or deregulated contractility, the disruption of microvascular integrity, and a hypoxia-induced imbalance in VEGF signaling. This imbalance leads to the uncontrolled development of fragile and highly permeable nascent blood vessels prone to hemorrhage and visual occlusion. Therapies that intervene in this inflammatory process must balance the beneficial effects of reduced neovascularization with the requirement not to harm the normal vasculature or the associated functional supportive epithelial and neuronal cells.

VEGF is an essential moderator of the angiogenic process and implicated in the development of diabetic retinopathy. Anti-VEGF therapy has been developed to reduce retinal neovascularization. The therapies that fall under the anti-VEGF umbrella have been shown to increase visual acuity in DME and reduce the development of nascent blood vessels in PDR when compared to the standard photocoagulation. Although effective, these therapies are not without fault. Most studies are small and suffer from a very focused design. Large, long-term prospective studies that involve close monitoring for adverse events will greatly advance the current research available. However, these studies may not be feasible in the context of pharmaceutical development and cost.

The adverse events that have emerged from current research raise some cause for concern. Particularly, a violation of the blood-retinal barrier by anti-VEGF agents can have catastrophic systemic effects. Further, the long-term effects of these agents are still unknown. VEGF plays such a critical role in the eye as well as systemically, simply inhibiting its action universally may cause significant adverse events after years of use.

Alternatives to photocoagulation and anti-VEGF injections are being investigated. They include therapies that target the mechanisms behind pericyte apoptosis and detachment. TNF-*α* inhibition could reduce endothelial damage and pericyte apoptosis [15]. Inhibition of the *β*IG-H3 protein could also serve as a future target [18]. Targeted regulation of angiopoietin isoforms in the Ang/Tie system may also offer a therapeutic approach to diabetic complications.

The use of corticosteroids has also emerged as a potential treatment targeting the inflammatory cascade. Clinical trials of intravitreal triamcinolone for patients with diabetic macular edema have shown little long-term benefit relative to focal/grid photocoagulation [114]. The trials have also indicated the significant presence of adverse events such as increased intraocular pressure and increased incidence of cataracts. Ozurdex (*Allergan)*, an intravitreally injectable dexamethasone implant, has shown beneficial effects in the treatment of DME [115]. Further noninferiority trials are necessary before widespread adoption.

Although further investigation into the contractility apparatus of the pericyte endothelial interaction is necessary, it presents a novel target for therapeutic intervention. Future developments that act on the calpain-dependant cleavage of talin as an integral step in pericyte contractility could preferentially eliminate the need for anti-VEGF intervention.

Alternatively, recent research investigating hemangioma-derived stem cells (HemSCs) has shown differentiation of HemSCs to pericytes through the NOTCH family ligand JAGGED1. This represents original evidence for a human postnatal vascular progenitor cell [116]. Although significant further research is needed, injecting HemSCs into a retinopathic eye can bestow the stability and regulation of healthy pericytes in place of ones damaged by hyperglycemia.

Additional current reviews highlight emerging therapeutic possibilities and future areas of research [117, 118].

The retinal complications that arise from chronic hyperglycemia have a significant impact on the lives of diabetic patients. Amelioration of these complications and their damaging effects will greatly benefit these patients and impact overall healthcare. Anti-VEGF therapies inhibit an essential mediator in the DR cascade and have been effective in causing regression of neovascularization and improving visual acuity. However, the evidence shows that complete inhibition of VEGF isoforms leads to known adverse events and unknown systemic and long-term consequences. We urge caution at the use of anti-VEGF agents for DME and PDR because the safety and efficacy of these drugs has yet to be fully established. Larger controlled trials are necessary as the vetting process continues. Also essential is the development of alternative therapies that target the pathogenic process leading to neovascularization. Focused therapies involving pericyte-endothelial-cell contractile apparatus and HemSCs are novel avenues with great potential. Due to the complexity of angiogenesis in DR, no one therapy will likely be perfect. However continued research into the pathogenesis and mechanical mechanisms behind this vascular insufficiency will highlight innovative opportunities for intervention.

## Figures and Tables

**Table 1:** Possible causes of pericyte dropout.

Factors	Evidence
BAX expression	(i) Proapoptotic member of the Bcl-2 family [12].
	(ii) Increased levels shown in retinal pericyte nuclei [13].
	(iii) Shift of the balance toward pericyte apoptosis.

TNF-*α*	(i) Cytokine involved in the regulation of immune cells during systematic inflammation including apoptosis [14].
	(ii) Elevated levels of TNF-*α* are associated with the early events of DR [14].
	(iii) Inhibition of TNF-*α* shown to cause a large reduction in the number of microvascular cells that expressed apoptotic indicators [15].

TGF-*β*	(i) Cytokine that regulates signaling pathways.
	(ii) High concentrations evidenced in response to hyperglycemia in pericytes and other vascular cells [16, 17].
	(iii) Increased TGF-*β* leads to *β*IG-H3 and RGD signaling that activates the capsase signaling pathway leading to apoptosis [18–23].

Ang/Tie	(i) Ang-2/Tie-2 binding shown to produce downstream pericyte apoptosis [24–26].
	(ii) Ang-2 is upregulated in retinal pericytes in response to hyperglycemia [27].

**Table 2:** Clinical trials of anti-VEGF pharmaceuticals for diabetic macular edema.

Drug/intervention	Status/paper	Design	*N*	Follow-up	Population	Author conclusions
Intravitreal pegaptanib versus sham injections	Cunningham Jr. et al. [28]	Randomized; double masked; Dose-ranging; controlled	172	36 weeks	Center involving DME, VA 20/50–20/320	Pegaptanib group had better VA, reduction in CRT, and less likely to need photocoagulation at followup

Intravitreal pegaptanib versus sham injections	Sultan et al. [29]	Randomized; sham controlled; multicenter; parallel group	260 in year 1 207 in year 2	2 years	Center involving DME	Pegaptanib offers clinical benefit for patients with DME: better VA, reduced CRT

Comparing laser alone, laser with intravitreal triamcinolone, laser with intravitreal ranibizumab, and intravitreal ranibizumab alone	Active, no publication (NCT00444600)	Randomized; double masked; parallel assignment; four treatment arms	691	22 months	Center involving DME	Results not yet published

ranibizumab versus nontreatment	Massin et al. [30] (RESOLVE)	Randomized; double masked; parallel assignment	100	12 months	Center involving DME	Ranibizumab is effective in improving BCVA and is well tolerated in DME

Ranibizumab with laser versus laser alone	Mitchell et al. [31] (RESTORE)	Randomized; double Masked; laser controlled; multicenter	345	12 months	Type 1 and 2 diabetic patients with visual impairment due to DME	Combined therapy provided superior VA gain. No difference detected at 1 year

Ranibizumab versus laser	Active, no publication (LUCIDATE) (NCT01223612)	Randomized; open label; parallel assignment	40	48 weeks	Type 1 and type 2 diabetic patients with DME	Results not yet published

Intravitreal injection on bevacizumab (4 doses) versus focal photocoagulation	Scott et al. [32]	Randomized; partially masked; five treatment arms	121	24 weeks	Center involving DME	Promising data warranting a phase III trial

Intravitreal bevacizumab versus triamcinolone	Completed, no publication (NCT01342159)	Randomized; single blind; parallel assignment; three treatment arms	80	20 months	Center involving DME	Results not yet published

Intravitreal bevacizumab dose comparison	Lam et al. [33]	Randomized; dose ranging	52	62 weeks	Diffuse, center involving DME	Both treatment arms were associated with similar reduced CRT and increased BCVA

Intravitreal bevacizumab alone or in combination with intravitreal triamcinolone versus macular laser photocoagulation	Soheilian et al. [34]	Randomized; double masked; three treatment arms	150	24 weeks	Clinically significant DME	Bevacizumab arm yielded a better visual outcome versus photocoagulation

This list, while comprehensive, is not exhaustive. Current clinical trial information can be found at http://www.clinicaltrials.gov/

*N*: Number of eyes; DME: diabetic macular edema; VA: visual Acuity; CRT: central retinal thickness; BCVA: best corrected visual acuity.
